# BAG3 regulates formation of the SNARE complex and insulin secretion

**DOI:** 10.1038/cddis.2015.53

**Published:** 2015-03-12

**Authors:** V Iorio, M Festa, A Rosati, M Hahne, C Tiberti, M Capunzo, V De Laurenzi, M C Turco

**Affiliations:** 1 Department of Pharmacy, University of Salerno, via Giovanni Paolo II, 132, Fisciano, SA, Italy; 2 BIOUNIVERSA S.r.l., University of Salerno, via Giovanni Paolo II, 132, Fisciano, SA, Italy; 3 Institut de Génétique Moléculaire de Montpellier, CNRS UMR5535, Montpellier, France; 4 Department of Clinical Sciences, University of Rome Sapienza, Rome, Italy; 5 Department of Medicine and Surgery, University of Salerno, Via S. Allende, Baronissi, SA, Italy; 6 Department of Experimental and Clinical Sciences, University G. D'Annunzio and Fondazione G. D'Annunzio, Ce.S.I., Chieti, Italy

## Abstract

Insulin release in response to glucose stimulation requires exocytosis of insulin-containing granules. Glucose stimulation of beta cells leads to focal adhesion kinase (FAK) phosphorylation, which acts on the Rho family proteins (Rho, Rac and Cdc42) that direct F-actin remodeling. This process requires docking and fusion of secretory vesicles to the release sites at the plasma membrane and is a complex mechanism that is mediated by SNAREs. This transiently disrupts the F-actin barrier and allows the redistribution of the insulin-containing granules to more peripheral regions of the *β* cell, hence facilitating insulin secretion. In this manuscript, we show for the first time that BAG3 plays an important role in this process. We show that BAG3 downregulation results in increased insulin secretion in response to glucose stimulation and in disruption of the F-actin network. Moreover, we show that BAG3 binds to SNAP-25 and syntaxin-1, two components of the t-SNARE complex preventing the interaction between SNAP-25 and syntaxin-1. Upon glucose stimulation BAG3 is phosphorylated by FAK and dissociates from SNAP-25 allowing the formation of the SNARE complex, destabilization of the F-actin network and insulin release.

Bcl2-associated athanogene 3 (BAG3) protein is a member of BAG family of co-chaperones that interacts with the ATPase domain of the heat shock protein (Hsp) 70 through a specific domain known as BAG domain.^1^ In addition to this structural domain, BAG3 also contains a WW domain that is a protein interaction module that binds to the proline-rich motif, XPPXY^1, 2^ and a proline-rich domain (PXXP) that modulates the interaction with SH3 domain-containing protein. These domains have been identified in a variety of signal transduction proteins that interact with plasma membrane receptor complexes or with components of the submembranous cytoskeleton^3^ suggesting that BAG3 might be a chaperone or a regulatory protein for proteins involved in cell migration and/or adhesion.^4, 5, 6, 7, 8^ Two BAG3 forms have been described so far: one is the full-length product of the *bag3* gene having an apparent mass of 74 kDa; the other one is a shorter form found in association to neural synaptosomes.^4, 9^ The full-length protein is normally localized in the cytoplasm and is mainly concentrated in the rough endoplasmic reticulum. Upon cell exposure to stressors, a slightly different molecular weight band can be observed and the protein runs as a doublet in a standard western blot.^10^ The origin of this doublet is currently unknown, but it possibly derives from post-translational modifications such as phosphorylations, indeed BAG3 protein contains several serine-rich motifs and 10 tyrosine residues that could be kinase targets. Tyrosine phosphorylation of BAG3 has been reported upon EGF stimulation of human breast cancer cell lines,^11^ furthermore, recently, it was shown that PKC mediates phosphorylation of BAG3 at Ser187 in tyroid cancer cells.^12^ BAG3 is constitutively expressed in myocytes and a few other normal cell types, while its expression can be induced by a variety of stressful stimuli in most cell types.^4^ In contrast BAG3 is constitutively expressed in several tumors and cancer cell lines where it has been shown to play an anti-apoptotic role.^4, 10, 13, 14^ Recently, we found that BAG3 is overexpressed in pancreatic ductal adenocarcinoma, where it is involved in sustaining pancreatic cancer cell survival. Furthermore, in this study we found a moderate positivity of BAG3 in the islets of Langerhans of pancreatic adenocarcinoma (PDAC) patients, whereas normal pancreatic ducts and pancreatic acinar cells exhibited no BAG3 expression.^15^ In response to elevated blood-glucose levels, pancreatic-islet *β*-cells release insulin in order to maintain glucose homeostasis. Dysfunction of this secretory response is considered to be one of the causal factors in the etiology of type 2 diabetes mellitus.^16^ Insulin, stored in large dense-core granules in pancreatic beta cells, is released in two phases by exocytosis following glucose stimulation.^17^ The first phase results in a transient peak of secretion lasting only a few minutes, whereas the second phase maintains a lower but persistent secretion rate.^18, 19^ The first phase is due to the triggering of the ATP-sensitive K(+) (K(ATP)) channel-dependent pathway that increases [Ca(2+)](i) and has been thought to discharge the granules from a ‘readily releasable pool'.^20, 21, 22, 23, 24^ The second phase of insulin secretion requires transport of the reserve granules pool to the plasma membrane^25, 26^ and involves actin cytoskeleton remodeling.^27, 28^ Insulin granule exocytosis requires docking and fusion of secretory vesicles to the release sites at the plasma membrane. This is mediated by a core machinery of membrane-associated soluble *N*-ethylmaleimide-sensitive factor attachment protein receptors (SNAREs), which can be classified into two subfamilies: vesicle SNAREs (v-SNAREs) found on the vesicles and target SNAREs (t-SNAREs) found on the target membranes.^29^ In *β*-cells the v-SNARE protein VAMP-2 is known to interact specifically with the t-SNARE protein syntaxin-1 and the synaptosome-associated protein of 25 kDa (SNAP-25) upon trafficking of a vesicle to a target membrane, bringing the two membranes into close proximity to allow fusion.^30, 31, 32^ Earlier studies have demonstrated that isolated insulin-containing granules co-sediment with filamentous actin (F-actin),^33^ which is organized as a dense web beneath the plasma membrane, blocking access of secretory vescicles to the t-SNARE complex at cell membrane.^33, 34, 35, 36^ Glucose stimulation of beta cells leads to focal adhesion kinase (FAK) phosphorylation, which acts on the Rho family proteins (Rho, Rac and Cdc42) that direct F-actin remodeling.^28, 37^ This transiently disrupts the F-actin barrier and allows the redistribution of the insulin-containing granules to more peripheral regions of the *β*-cell,^36, 38^ hence facilitating insulin secretion. It has been shown that BAG3 is involved in actin folding.^6, 39, 40, 41, 42^ It was also reported that BAG3 may negatively regulate adhesion, focal adhesion assembly, signaling and migration via its PXXP domain.^7, 43^ These data suggest that in *β*-cells BAG3 could play a role in insulin release regulating intracellular trafficking or the exocytosis mechanism. Indeed using a mouse insulinoma *β*-TC-6 cell model here we show that BAG3 silencing affects F-actin polymerization state resulting in increased insulin secretion. Moreover, we show that BAG3 physically interacts with components of the SNARE complexes: SNAP-25/syntaxin-1, and is phosphorylated by FAK upon glucose stimulation. Our data suggest a model by which the activation of FAK after glucose entry, increases BAG3 tyrosine phosphorylation with subsequent loss of the BAG3/ SNAP-25 interaction, thus allowing the formation of the t-SNARE complex and exocytosis of insulin vesicles.

## Results

### BAG3 is highly expressed in human islet of Langerhans and co-localizes with insulin

**Figure 1:**
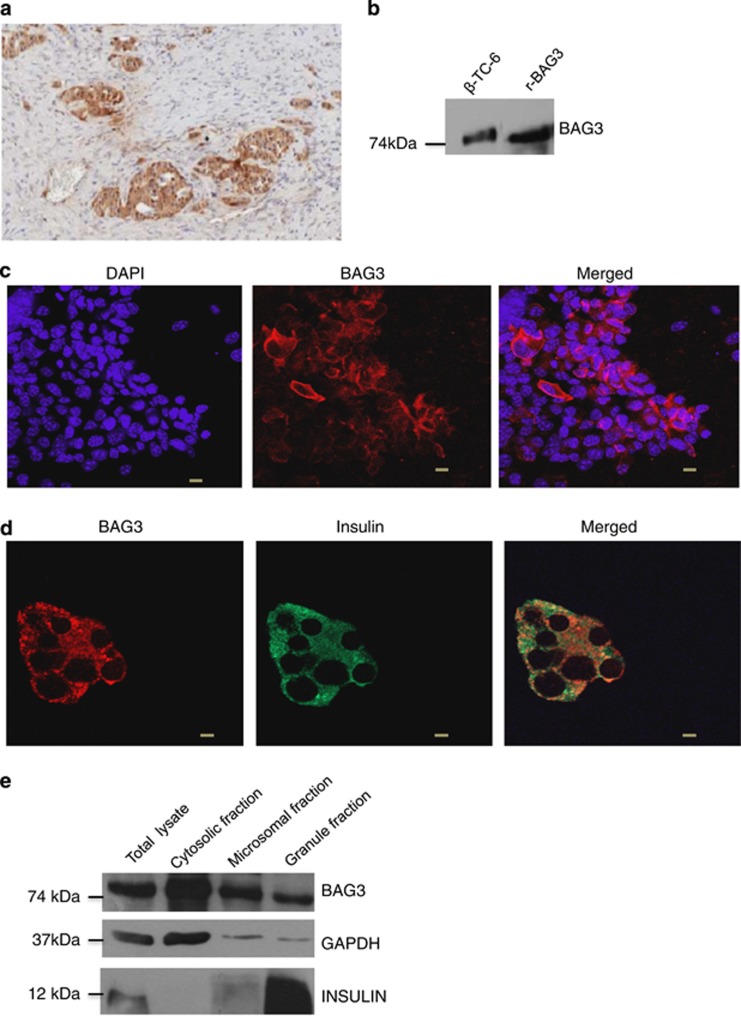
BAG3 is expressed in pancreatic beta cells. (**a**) A representative section of human pancreas showing high expression of BAG3 in the pancreatic islets compared with the exocrine tissue. (**b**) Western blot of *β*-TC-6 lysate and BAG3 recombinant incubated with a polyclonal antibody against BAG3. (**c**) *β*-TC-6 cells were fixed and stained with a monoclonal anti-BAG3 antibody (red) followed by incubation with fluorescein-conjugated secondary antibody. DAPI was used to stain cell nuclei (scale bars, 10 *μ*m). (**d**) *β*-TC-6 cells were fixed and stained with a monoclonal anti-BAG3 antibody (red) and with an anti-insulin antibody (green) followed by incubation with fluorescein-conjugated secondary antibodies. Yellow regions indicate co-localization (scale bars, 10 *μ*m; zoom 2). All images are fully representative of three independent experiments. (**e**) *β*-TC-6 cells were fractionated into cytosolic, microsomal and insulin granule fraction; 15 *μ*g of each sample was loaded for immunoblotting with anti-BAG3 antibody. Anti-GAPDH and anti-insulin antibody were used to confirm the purity of isolated subcellular fractions. These results are representative of two independent experiments

While studying expression of BAG3 in PDACs we noticed that pancreatic islets in normal pancreatic tissue surrounding the tumor strongly stained for BAG3, we therefore decided to further investigate the role of this protein in endocrine pancreatic cells. Indeed we confirmed this finding in additional pancreas sections and as shown in Figure 1a, BAG3 shows high expression in pancreatic islets, while the exocrine portion of the pancreas is as expected negative. We then chose to use an established mouse insulinoma cell line, *β*-TC-6, to further investigate the role played by BAG3 in endocrine pancreatic cells. As shown in the western blot in Figure 1b this cell line does express BAG3 that is localized in the cytoplasm of these cells (Figure 1c). Interestingly, BAG3 appears to co-localize with insulin-containing granules as shown by co-immunostaining (Figure 1d) and by subcellular fractionation (Figure 1e) that shows the presence of BAG3 in the insulin granule fraction.

### BAG3 knockdown decreases insulin cell content and increases its secretion

**Figure 2:**
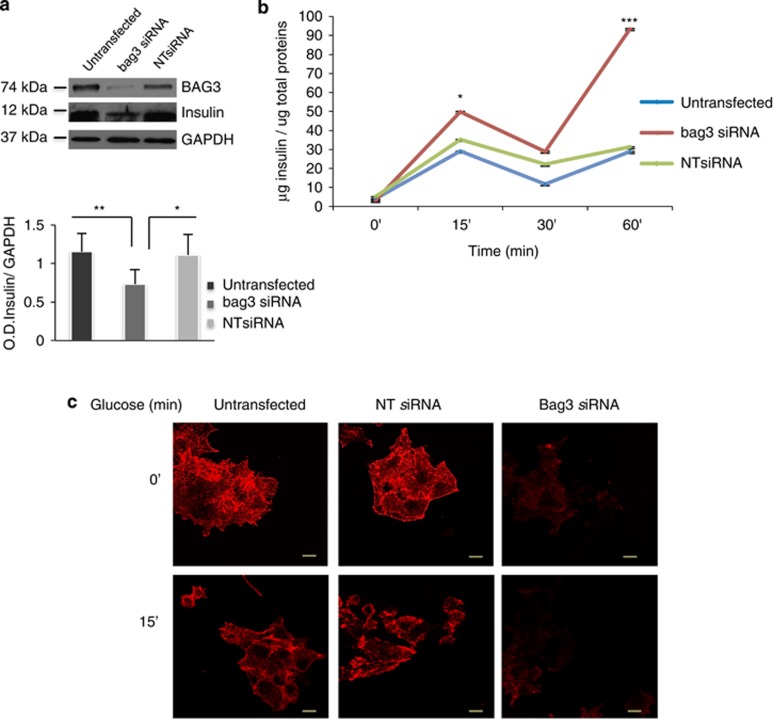
BAG3 downregulation decreases insulin content levels and potentiates glucose-stimulated insulin secretion. (**a**) *β*-TC-6 cells at 80% confluence were transfected with a bag3-specific small interfering (si) RNA or a non-target (si) RNA (NT siRNA). After 48 h, whole-cell extracts were obtained and analyzed by western blot with an anti-BAG3 polyclonal antibody, anti-insulin antibody or, as a loading control, an anti-GAPDH monoclonal antibody. Densitometric analysis of insulin expressed as insulin/GAPDH ratios is reported in the lower panel. (**b**) Insulin secretory response of *β*-TC-6 cells transfected with a bag3-specific siRNA or an NT siRNA. The insulin secreted amount was evaluated by ELISA test on *β*-TC-6 supernatants collected at 15, 30 and 60 min after glucose stimulation. Time 0 represents insulin levels after starvation before addition of glucose. Data are mean±S.E.M. (*n*=2). *P<0.05, **P<0.01 and ***P<0.001. (**c**) *β*-TC-6 cells were transfected with a bag3-specific siRNA or an NT siRNA; 48 h after transfection cells were stimulated for 15 min with 25-mM glucose, fixed and stained with TRITC-conjugated phalloidin. All pictures are fully representative of multiple images from two independent experiments (scale bars, 10 *μ*m)

We then tested the possibility that BAG3 influenced insulin levels and secretion. As shown in Figure 2a silencing BAG3 in *β*-TC-6 cells results in a significant reduction of their insulin content. Moreover, evaluation of insulin secretion in the supernatant by ELISA, following stimulation with 25-mM glucose, shows that this is strongly affected by BAG3 silencing. Under these experimental conditions there are two secretion peeks, one after 15 min and the other after 60 min (Figure 2b).^44^ BAG3 silencing results in increase of both early and late secretion. Similar results were obtained using a different siRNA to silence BAG3 excluding off-target effects (Supplementary Figure 1A).

### BAG3 knockdown impedes F-actin polymerization

It has recently been shown that localized F-actin remodeling is a requisite for the normal biphasic pattern of nutrient-stimulated insulin secretion.^37^ F-actin constitutes a barrier that prevents docking and fusion of insulin granules to the plasma membrane allowing insulin exocytosis,^16^ in fact latrunculin B, a potent agent that binds sequestrates actin monomers thus preventing F-actin polymerization, markedly potentiates glucose-stimulated insulin secretion in a *β*-cell line.^38, 45^ We therefore investigated the possibility that BAG3 affected insulin secretion by altering F-actin remodeling. To this end, we performed confocal experiments on *β*-TC-6 transfected with *bag3* siRNA, using TRITC-conjugated phalloidin to visualized F-actin. As shown in Figure 2c and Supplementary Figure 1B, the actin cytoskeleton appears to be intact in the control cells, and after 15 min of glucose stimulation a redistribution of actin fibers becomes visible. This is consistent with the evidences that glucose induces F-actin depolymerization.^35, 36^ Cells treated with NT siRNA revealed a similar pattern. Conversely, cells treated with *bag3* siRNA display a clear disappearance of phalloidin staining, both in the unstimulated cells as well as in the cells stimulated with glucose, suggesting a failure in F-actin polymerization in BAG3-deficient *β*-cells.

### BAG3 interacts with t-SNARE complex: SNAP-25/syntaxin-1

**Figure 3:**
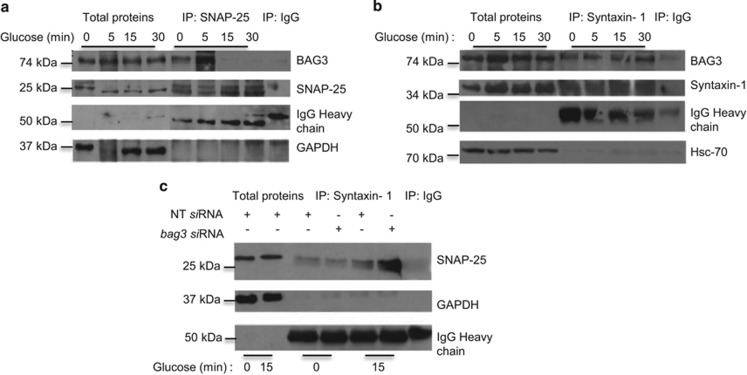
BAG3 associates with SNAP-25 and syntaxin-1 and regulates the formation of the SNARE complex. *β*-TC-6 cells were stimulated with 25-mM glucose for the indicated time. Clarified whole-cell detergent extracts were prepared and immunoprecipitated with (**a**) anti-SNAP-25 or (**b**) anti-syntaxin-1 antibodies. Immune precipitates were analyzed by western blot with anti-SNAP-25, syntaxin-1, BAG3, Hsc-70 and GAPDH antibodies. Control immunoprecipitations were performed in parallel using mouse IgG. These results are representative of three sets of lysates prepared from independent experiments. (**c**) *β*-TC- 6 cells at 80% confluence were transfected with a bag3-specific small interfering (si) RNA or a non-target (si) RNA (NT siRNA); 48 h after transfection, cells were stimulated with 25-mM glucose for 15 min. Clarified whole-cell detergent extracts were prepared and immunoprecipitated with anti-syntaxin-1. Immune precipitates were subjected to western blot with, anti-SNAP-25 and GAPDH antibodies. Control immunoprecipitations were performed in a similar manner, using mouse IgG. These results are representative of two independent experiments

Cortical F-actin remodeling is known to couple granule mobilization to the SNARE exocytosis machinery.^38, 45, 46^ The formation of the t-SNARE complex by the interaction of SNAP-25 with syntaxin-1 at the plasma membrane is essential for the pairing with the vesicle v-SNARE complex.^31, 47, 48^ It is therefore possible that BAG3 affects F-actin remodeling by interacting with components of the SNARE complex. To test this hypothesis, we performed co-immunoprecipitation experiments in basal conditions and after 5, 15 and 30 min of glucose stimulation. *β*-TC-6 cell extracts were immunoprecipitated using anti-SNAP-25 (Figure 3a) and anti-syntaxin-1 (Figure 3b) antibodies and revealed using a polyclonal anti-BAG3 antibody. Interestingly, while BAG3 is pulled down with the antibody against SNAP-25 at basal levels and even more after 5 min of glucose stimulation, this interaction is lost at longer time points (Figure 3a). On the contrary BAG3 appears to interact with syntaxin-1 with the same intensity throughout the stimulation (Figure 3b).

We then investigated the possibility that BAG3 affected the interaction between syntaxin-1 and SNAP-25 and thus the fusion to the plasma membrane and exocytosis of insulin vesicles. As shown in Figure 3c treatment with glucose 25 mM for 15 min results in increased interaction between these two proteins, however, in the absence of BAG3 this interaction is far more pronounced following glucose stimulation. These data strongly suggest that BAG3 is an important regulator of the syntaxin-1/SNAP-25 complex and prevents its formation in basal conditions, while under glucose stimulation BAG3 does not bind SNAP-25 allowing it to bind syntaxin-1 thus promoting exocytosis.

### FAK controls BAG3 tyrosine phosphorylation

**Figure 4:**
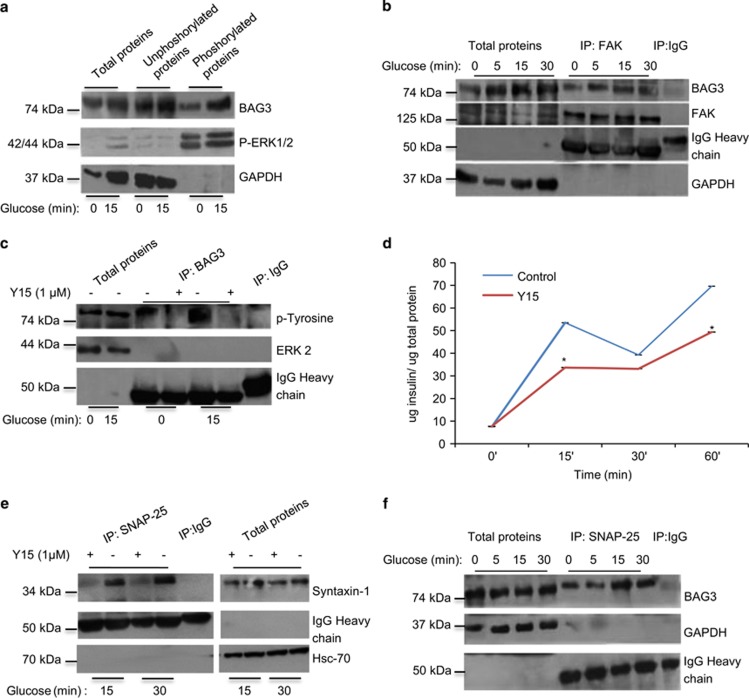
BAG3 tyrosine phosphorylation by FAK disrupts the interaction between BAG3 and SNAP-25. (**a**) *β*-TC-6 cells were stimulated with 25-mM glucose for 15 min. Phosphorylated proteins were separated from unphosphorylated proteins using affinity chromatography. Then 20 *μ*g of each sample was analyzed by western blot with anti-BAG3 antibody. Anti-p-ERK1/2 antibody was used as control of the phosphorylated protein fraction and anti-GAPDH antibody as control of the unphosphorylated fraction. These results are representative of two independent experiments. (**b**) *β*-TC-6 cells were stimulated with 25-mM glucose from 5–30 min. Clarified whole-cell detergent extracts were immunoprecipitated with an anti-FAK antibody. Immune precipitates were analyzed by western blot using anti-BAG3, FAK and GAPDH antibodies. Control immunoprecipitations were performed similarly using mouse IgG. These results are representative of three independent experiments. (**c**) *β*-TC-6 cells were stimulated with 25-mM glucose for 15 min with or without the FAK inhibitor Y15. Clarified whole-cell detergent extracts were immunoprecipitated with an anti-BAG3. Immune precipitates were analyzed by western blot using anti-phosphotyrosine and anti-ERK2 antibodies. Control immunoprecipitations were performed in parallel using mouse IgGs. These results are representative of three independent experiments. (**d**) Insulin secretory response of *β*-TC-6 cells treated with FAK inhibitor Y15. Insulin levels were evaluated by ELISA on *β-*TC-6 supernatants collected 15, 30 and 60 min after glucose stimulation. Data are mean±S.E.M. (*n*=2). *P<0.05. (**e**) *β*-TC-6 cells were stimulated with 25-mM glucose for 15 and 30 min with the FAK inhibitor Y15. Clarified whole-cell detergent extracts were immunoprecipitated with an anti-SNAP-25 antibody. Immune precipitates were analyzed by western blot with anti-syntaxin-1 and Hsc-70 antibodies. Control immunoprecipitations were performed in parallel using mouse IgG. (**f**) *β*-TC-6 cells were stimulated with 25-mM glucose for 5–30 min with the FAK inhibitor Y15. Clarified whole-cell detergent extracts were immunoprecipitated with an anti-SNAP-25 antibody. Immune precipitates were analyzed by western blot with anti-BAG3 and GAPDH antibodies. Control immunoprecipitations were performed in parallel using mouse IgG. These results are representative of two independent experiments

An essential part of the signaling leading to insulin secretion is FAK autophosphorylation following glucose stimulation that leads to phosphorylation of other substrate proteins and the formation of the multiproteic SNARE.^28, 49^ We therefore checked whether FAK could interact with BAG3 and phosphorylate it. As shown in Figure 4a upon glucose stimulation there is an increase in BAG3 in the fraction containing phosphorylated proteins. As shown in Figure 4b BAG3 can be immunoprecipitated with FAK both in basal conditions and upon stimulation with glucose. Moreover, BAG3 immunoprecipitation in basal conditions and after 15 min of glucose stimulation followed by western blotting using a monoclonal anti-tyrosine antibody (Figure 4c) shows that BAG3 is phosphorylated in tyrosine and that tyrosine phosphorylation increases upon glucose stimulation. Most importantly treatment with 1-*μ*M Y15 a FAK-specific inhibitor completely abrogates BAG3 phosphorylation, thus showing that BAG3 is FAK dependent and probably BAG3 is directly phosphorylated by FAK. As previously reported,^28^ under these experimental conditions, Y15 reduces insulin secretion (Figure 4d). Furthermore Y15 also reduces the interaction between SNAP-25 and syntaxin-1 (Figure 4e), and therefore SNARE complex formation. Finally, as shown in Figure 4f inhibiting BAG3 phosphorylation with the FAK inhibitor Y15 results in persistence of the interaction between BAG3 and SNAP-25 upon glucose stimulation.

## Discussion

**Figure 5:**
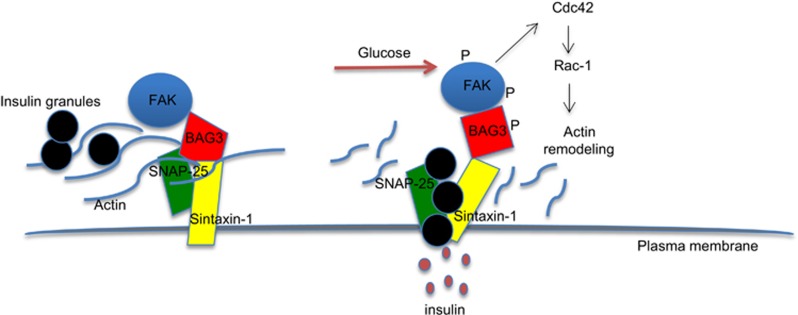
Schematic illustration of the proposed role of BAG3 in insulin granule exocytosis. In basal conditions F-actin barrier (presumably stabilized by BAG3) prevents insulin granule mobilization to the plasma membrane. FAK autophosphorylation, upon glucose stimulation, leads to activation of the Rho family proteins (Rac-1 and Cdc42) that direct F-actin remodeling and to phosphorylation of BAG3. Phosphorylated BAG3 no longer binds SNAP-25 allowing its interaction with syntaxin-1 and the formation of the t-SNARE complex, thus promoting insulin secretion

Insulin secretion by exocytosis of its storage granules is a complex mechanism that involves numerous proteins and is regulated by multiple pathways to finely control circulating glucose levels. Alteration of these pathways may contribute to the pathogenesis of type 2 diabetes.^16^ Here, we show that BAG3 is strongly expressed in islets of Langerhans *β*-cells where it appears to play a role in regulating insulin secretion. Indeed silencing BAG3 in *β*-TC-6 mouse insulinoma cells results in reduced intracellular content of insulin and in its increased secretion in response to glucose stimulation. In line with a number of reports showing that F-actin disruption removes a physical barrier and allows the redistribution of the insulin-containing granules to more peripheral regions of the *β*-cell, increasing insulin secretion,^28, 38, 45^ we found that silencing BAG3 results in disruption of the F-actin network both in basal conditions and than upon glucose stimulation. We also show that BAG3 binds to components of the t-SNARE complex SNAP-25 and syntaxin-1 preventing the interaction between these two proteins, thus the mature complex formation. Upon glucose stimulation binding between BAG3 and SNAP-25 is lost and this allows the binding between SNAP-25 and syntaxin-1, the mature complex can now interact with the v-SNARE complex promoting exocytosis. Finally, we show that BAG3 release from its interaction with SNAP-25 is due to BAG3 tyrosine phosphorylation. This phosphorylation is dependent on the activity of FAK, a tyrosine kinase that is activated by autophosphorylation in response to glucose stimulation.^46, 50, 51, 52, 53^ The phosphorylation of FAK appears to be one of the initial events in the signaling cascade leading to insulin secretion and its consequent activation determines the recruitment of other proteins to the adhesion complexes, and promotes actin remodeling finally resulting in insulin granule exocytosis.^49^ Deletion of FAK in mice B-cells results in impaired glucose-dependent insulin secretion, glucose intolerance and eventually in loss of *β*-cells, strongly suggesting that alteration of these pathways can play a role in diabetes pathogenesis. Consistently decreased phosphorylated FAK expression was observed in pancreatic sections from type 2 diabetes patients.^54^ In conclusion, based on our data we suggest that one of the FAK targets is BAG3 and that its phosphorylation allows the formation of the SNARE complex as well as promotes F-actin remodeling. In our model (Figure 5) upon glucose stimulation FAK is activated by autophosphorylation and phosphorylates a number of target proteins among which is BAG3. Phosphorylated BAG3 no longer binds SNAP-25 allowing its interaction with syntaxin-1 and the formation of the t-SNARE complex, thus promoting insulin secretion. At the same time possibly phosphorylated BAG3 is no longer capable of stabilising F-actin network again favoring the migration of the insulin granules to the plasma membrane and their exocytosis. Further studies will be required to elucidate the mechanism by which BAG3 affects F-actin polymerization and whether tyrosine phosphorylation affects this BAG3 function. Moreover, further studies should be performed to clarify whether BAG3 alterations can contribute to diabetes pathogenesis.

## Materials and Methods

### Cell culture

Cells of the established murine *β*-TC-6 cell line were purchased from Istituto Zooprofilattico Sperimentale della Lombardia ed Emilia Romagna (IZSLER, Lugo, Ra, Italy). Cells were grown in DMEM (LONZA Group Ltd, Basel, Switzerland) culture medium containing 15% FBS; 25, 12.5 or 2.8-mM glucose; and 1% streptomycin/penicillin at 37°C in a 5% CO_2_ atmosphere. The medium was changed every 48 h, and cells were passaged once weekly using standard trypsin-EDTA concentrations.

### Tissue samples

We analyzed tissue samples from tissue microarray TMA PA2082 (US Biomax, Inc., Rockville, MD, USA) that contained normal pancreas tissue samples from 11 donors (6 men and 5 women; mean±S.D. age: 44.6±19.3 years).

### Antibodies and reagents

Antibodies recognizing ERK2 (sc-154), GAPDH (sc-32233), insulin (sc-9168), FAK (sc-271195) and Hsc-70 (sc-7298) were obtained from Santa Cruz Biotechnology, Inc. (Santa Cruz, CA, USA); SNAP-25 (mouse monoclonal) and syntaxin-1 (rabbit polyclonal) from Synaptic Systems (Gottingen, Germany); SNAP-25 (C-term; rabbit polyclonal) and syntaxin-1 (mouse monoclonal) from Antibodies-online (Aachen, Germany); p-tyrosine (# 9411), p-ERK1/2 (Thr202/Thr 404; # 9101) from Cell Signaling Technology, Inc. (Danvers, MA, USA). Anti-BAG3 antibody TOS-2 (rabbit polyclonal) and AC-1 (mouse monoclonal) were purchased from BIOUNIVERSA s.r.l. (Fisciano, SA, Italy). Mouse IgG isotype control (ABIN 398652) was obtained from Antibodies-online. FAK inhibitor 1,2,4,5-benzenetetraamine tetrahydrochloride (Y15) was obtained from Sigma-Aldrich (St. Louis, MO, USA). Phalloidin conjugates TRITC (P 1951) was purchased from Sigma-Aldrich. Phosphoprotein purification kit (37101) was obtained from Qiagen (Venlo, Limburg, Netherlands).

### Glucose-stimulated insulin secretion

*β* -TC-6 cells were plated in six-well plates at a density of 2.5 × 10^5^ in DMEM with 2.8-mM glucose. At the beginning of the third day of subculture, cells were incubated once for 30 min at 37°C in Krebs-Ringer bicarbonate buffer (118.5-mmol/l NaCl, 2.54-mmol/l CaCl_2_ 2H_2_O, 1.19-mmol/l KH_2_PO_4_, 4.74-mmol/l KCl, 25-mmol/l NaHCO_3_, 1.19-mmol/l MgSO_4_ 7H_2_O, 10-mmol/l HEPES (LONZA Group Ltd) and 0.1% bovine serum albumin (BSA), pH 7.4)(Sigma-Aldrich) with 2.8-mM glucose in a total volume of 1 ml. Cells were then stimulated with 25-mM glucose for 1 h. Media was collected at 15, 30 and 60 min after glucose stimulation, spun for 2 min at 13 000 r.p.m. and used to determine insulin concentration. Insulin secretion level was measured by ELISA (Mouse insulin ELISA kit; Mercodia, Sylveniusgatan, Uppsala, Sweden). Cells were then washed twice with PBS and lysed as described below. Protein concentration was determined in each sample by Bradford assay (Bio-Rad, Hercules, CA, USA). Total released insulin was normalized with protein concentration. Significance was determined by unpaired Student's test. *P=0.05 to 0.01, **P=0.01 to 0.001 and ***P<0.001.

### Western blot

Cells were harvested and washed twice with PBS 1 × solution (Mediatech, Herndon, VA, USA). Total proteins were extracted in buffer Nonidet P-40 (25-mM Tris, pH 7.4; 1% Nonidet P-40 (Sigma-Aldrich); 10% glycerol; 50-mM NaF; 10-mM Na-pyrophosphate; 137-mM NaCl; and 1-mM PMSF) supplemented with a protease inhibitor cocktail (Sigma-Aldrich), using three cycles of freeze and thaw. The lysates were centrifuged at 15 000 × *g* at 4°C and the soluble fractions were collected. Protein amount was determined by Bradford assay. Equal amounts of total protein (10 or15 *μ*g) from each sample were separated electrophoretically in 8, 10 or 15% SDS-PAGE gels and were blotted on a nitrocellulose membrane (Hybond; Amersham Life Sciences, St Louis, MO, USA). Nitrocellulose blots were blocked with 10% non-fat dry milk or 10% BSA in TBST buffer (20-mM Tris-HCl, pH 7.4; 500-mM NaCl; and 0.1% Tween 20, Sigma-Aldrich) and incubated with primary antibodies in TBST containing 5% BSA or 10% non-fat dry milk, overnight at 4°C. Immunoreactivity was detected by sequential incubation with horseradish peroxidase-conjugated secondary antibodies purchased from Pierce (Rockford, IL, USA) and ECL detection reagents purchased from Santa Cruz Biotechnology, inc. Scanning densitometry of the bands was performed using ImageJ software (1.47 V; Wayne Rasband, NIH, USA; http://imagej.nih.gov./ij). Significance was determined by unpaired Student's test. *P=0.05 to 0.01, **P=0.01 to 0.001 and ***P<0.001.

### Transfection of small interfering RNA

The bag3 siRNA (5′-AAGGUUCAGACCAUCUUGGAA-3′), bag3 siRNA b (5′-ATCGAAGAGTATTTGACCAAA-3′) and NT siRNA (5′-CAGUCGCGUUUGCGACUGG-3′) were synthesized by MWG Biotech (Ebersberg, Germany). *β*-TC-6 cells were transfected with a final siRNA concentration of 100 nM using Hiperfect transfection reagent (Qiagen) and then seeded in six-well plates for 48 h. Transfection efficiency was evaluated in each experiment by western blot analysis.

### Confocal microscopy

Cells were cultured on coverslips in six-well plates to 60–70% confluence. At third day of culture, coverslips were washed in PBS 1x and fixed in 3.7% formaldehyde in PBS 1 × for 30 min at room temperature, and then incubated for 5 min with PBS 1x 0.1-M glycine. After washing, coverslips were permeabilized with 0.1% Triton X-100 for 5 min, washed again and incubated with blocking solution (5% normal goat serum in PBS 1 × ) for 1 h at room temperature. Following incubation with a 1 : 100 dilution of anti-insulin polyclonal antibody, 3 *μ*g/ml of anti-BAG3 mouse monoclonal antibody AC-1 for 2 h at room temperature, coverslips were washed three times with PBS 1x. After incubation with a 1 : 500 dilution of goat anti-mouse IgG DyLigth 594-conjugated antibodies (Jackson ImmunoResearch, Baltimore, PA, USA) and a 1 : 500 dilution of goat anti-rabbit IgG DyLigth 488-conjugated antibodies (Jackson ImmunoResearch) at room temperature for 45 min, coverslips were again washed for three times in PBS. F-actin was visualized using a diluition 1 : 1000 of TRITC-conjugated phalloidin, 45 min at room temperature. Nuclei was visualized with a diluition 1 : 5000 of DAPI, 10 min at room temperature. The coverslips were washed once with PBS 1 × and once in distilled water and then mounted on a slide with interspaces containing 70% (v/v) glycerol. Samples were analyzed using a confocal laser scanning microscope (Zeiss LSM confocal microscope, Oberkochen, Germany). Images were acquired in sequential scan mode by using the same acquisition parameters (laser intensities, gain photomultipliers, pinhole aperture and objective x63 or x150) when comparing experimental and control material. For production of figures, brightness and contrast of images were adjusted by taking care to leave a light cellular fluorescence background for visual appreciation of the lowest fluorescence intensity features and to help comparison among the different experimental groups. Final figures were assembled using Adobe Photoshop 7 and Adobe Illustrator 10 (San Jose, CA, USA).

### Co-immunoprecipitation

BAG3, SNAP-25, syntaxin-1 and FAK proteins were immunoprecipitated from *β* TC-6 lysate using IP matrix mouse (sc-45042) purchased from Santa Cruz Biotechnology, inc. Briefly, 45 *μ*l of matrix were incubated with 3 *μ*g of anti-BAG3 (Ac-1), anti-SNAP-25, anti-syntaxin-1 or anti-FAK antibodies for 1 h at 4°C in continuous shaking. Then the matrix was washed with PBS 1x + 0.1% Tween twice and centifugated each time at 13 000 r.p.m. for 1 min at 4°C. Subsequently, 300 *μ*g of protein exctracts obtained from *β*-TC-6 lysates were loaded on the matrix and incubated for 2 h at 4°C by continuous shaking. After this incubation the matrix was washed with RIPA buffer (50-mM Tris-HCl, pH 7.6; 150-mM NaCl; 4-mM EDTA; 10-mM NaF; 10-mM Na-pyrophosphate; 1% NP-40; and 1% Na-deoxycholate) for three times and centifugated each time at 13 000 r.p.m. for 1 min at 4°C. Finally, the matrix was resuspended in 45 *μ*l of Laemmli buffer 2x and stored at −20°C. The samples were then processed for western blot.

### Immunohistochemistry

The immunohistochemistry protocol included deparaffinization in xylene, rehydration via decreasing concentrations of alcohol down to pure water, non-enzymatic antigen retrieval in citrate buffer (pH 6.0) for 30 min at 95°C, and endogenous peroxidase quenching with H_2_O_2_ in methanol for 20 min. After rinsing with PBS, the samples were blocked using 5% normal horse serum in 0.1% PBS or BSA. To detect BAG3, samples were incubated for 1 h at room temperature with the monoclonal antibody AC-1 at a concentration of 3 *μ*g/ml. After washing thoroughly with PBS, sections were incubated using a biotinylated secondary anti-mouse IgG for 20 min, then rinsed, incubated using avidin-biotin-peroxidase complex (Novocastra; Leica Microsystems, Milan, Italy) and developed using diaminobenzidine (Sigma-Aldrich). Finally, the sections were counterstained using hematoxylin, dehydrated in alcohol, cleared in xylene and mounted using Permount (Fisher Scientific Inc., Milan, Italy).

### Subcellular fractionation

Subcellular fractions of *β*-TC-6 cells were isolated as previously described.^55^ All procedures were performed on ice. Approximately, 4.5 × 10^7^ cells grown in 15-cm dishes were washed two times with cold PBS and scraped in 1 ml homogenization buffer (20-mM Tris-HCl, pH 7.4; 0.5-mM EDTA; 0.5-mM EGTA; 250-mM sucrose; and 1-mM dithiothreitol) containing the following protease inhibitors: leupeptin (10 g/ml), aprotinin (4 g/ml), pepstatin (2 g/ml), and PMSF (100 M). The cells were then disrupted by 10 strokes through a 27 G needle. The cell homogenates were centrifuged at 900 × *g* for 10 min to remove the nuclei and unbroken cells. Post-nuclear supernatant was centrifugated at 5500 × *g* for 15 min and then at 25 000 × *g* for 20 min to pellet the storage granule fraction. The supernatant was further centrifugated at 100 000 × *g* for 1 h to obtain the microsomial fraction (as pellet) and cytosolic fraction (supernatant). Protein amount in subcellular fractions was determined by Bradford assay.

## Abbreviations

BAG3: Bcl2-associated athanogene 3
SNARE: soluble *N*-ethylmaleimide-sensitive factor attachment protein receptor
FAK: focal adhesion kinase

## Notes

BIOUNIVERSA s.r.l., which produces anti-BAG3 antibodies, provided them free of charges for this study. MF, AR, VDL and MCT are shareholders of the company BIOUNIVERSA that provided some of the used antibodies. The remaining authors declare no conflict of interest.
