# Supplementary figures and images for: BAG3 regulates formation of the SNARE complex and insulin secretion
Source: Cell Death Dis. 2015 Mar 12;6(3):e1684–. doi: 10.1038/cddis.2015.53 (PMC4385931; doi:10.1038/cddis.2015.53)

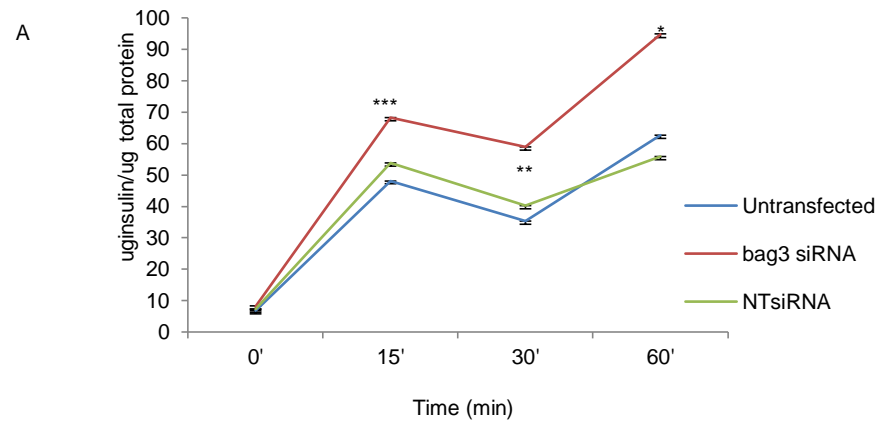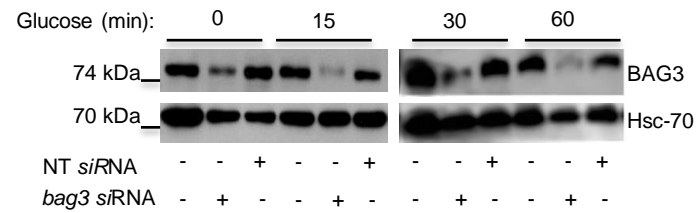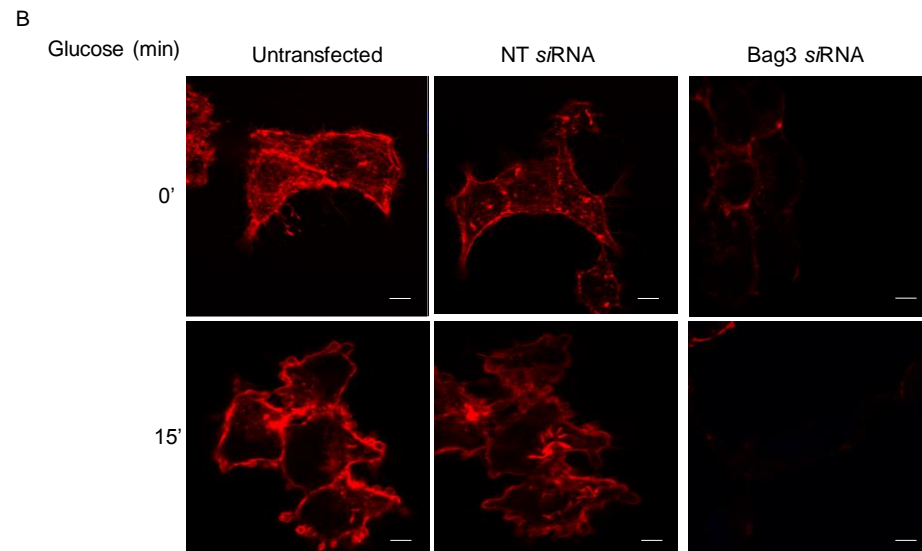

Supplement: Supplementary Figure 1 [file cddis201553x1.pdf]
