# Supplementary material for: BAG3 regulates formation of the SNARE complex and insulin secretion
Source: Cell Death Dis. 2015 Mar 12;6(3):e1684–. doi: 10.1038/cddis.2015.53 (PMC4385931; doi:10.1038/cddis.2015.53)
Supplement: Supplementary Information 1 [file cddis201553x2.docx]

**Legend to Supplementary Figure 1**

A) Insulin secretory response of β-TC -6 cells transfected with a bag3- specific small interfering (si) RNA (siRNA b ) or a non- target (si) RNA (NT *si*RNA). Insulin levels were evaluated by ELISA test on β –TC- 6 supernatants collected at 15, 30 and 60 minutes after glucose stimulation. Data are mean + SEM (n=2). *,P< 0.05, **,P < 0.01, ***, < 0.001. Pellets from cells were analyzed by western blot to confirm BAG3 silencing (lower panel). B) β-TC- 6 cells transfected with a bag3- specific small interfering (si) RNA or a non- target (si) RNA (NT *si*RNA). After 48 h cells were stimulated for 15 min with 25mM glucose, fixed and stained with TRITC conjugated phalloidin. All pictures are fully representative of multiple images from two independent experiments (scale bar = 5 μm).
